# Supplementary material for: Simultaneous Estimation of Gender Male and Atrial Fibrillation as Risk Factors for Adverse Outcomes Following Transcatheter Aortic Valve Implantation
Source: J Clin Med. 2020 Dec 7;9(12):3963. doi: 10.3390/jcm9123963 (PMC7762231; doi:10.3390/jcm9123963)
Supplement: Supplementary file 1 [file jcm-09-03963-s001.pdf]

SUPPLEMENTAL MATERIAL

Supplementary Table 1. In-hospital adverse events after TAVI in groups defined by gender and AF.

	**Overall n=1088**	**Female, AF(-) n=559**	**Male, AF(-) n=266**	**Female, AF(+) n=187**	**Male, AF(+) n=76**	**p-value**
All-cause death	13, 1.2%	2, 0.4%	6, 2.3%	4, 2.1%	1, 1.3%	0.06
Life-threatening + Major bleeding	77, 7.1%	40, 7.2%	19, 7.1%	15, 8.0%	3, 3.9%	0.73
**Acute kidney injury**	59, 5.4%	**20, 3.6%**	**17, 6.4%**	**14, 7.5%**	**8, 10.5%**	**0.02**
Major vascular complications	31, 2.8%	19, 3.4%	6, 2.3%	5, 2.7%	1, 1.3%	0.67
Disabling stroke	15, 1.4%	5, 0.9%	4, 1.5%	4, 2.1%	2, 2.6%	0.44
All events combined	150, 13.8	63, 11.3%	42, 15.8%	32, 17.1%	13, 17.1%	0.09

Supplementary Table 2. Univariate Cox proportional hazard analyses for predictors of all–cause mortality following TAVI.

	HR	95% CI	*p*-value
Age (per 1 year older)	1.02	0.99–1.05	0.15
BMI (per 1 kg/m^2 higher)	0.93	0.89–0.97	**0.001**
NYHA class (per 1 higher)	1.78	1.40–2.26	**<0.001**
Hypertension	0.87	0.62–1.23	0.44
Diabetes mellitus	1.20	0.87–1.66	0.26
Dyslipidemia	0.72	0.54–0.97	**0.03**
History of heart failure	2.42	1.81–3.24	**<0.001**
History of cancer	1.57	1.13–2.17	**0.007**
History of stroke	1.24	0.80–1.92	0.34
COPD	2.23	1.49–3.34	**<0.001**
PAD	1.62	1.15–2.28	**0.006**
CAD	1.19	0.87–1.61	0.28
History of coronary revascularization	1.23	0.89–1.71	0.21
Steroid	1.46	0.91–2.36	0.12
Antiplatelets	0.93	0.69–1.24	0.61
Anticoagulants	2.00	1.48–2.70	**<0.001**
Antithrombotic agents	1.43	1.01–2.04	**0.046**
Beta-blockers	1.30	0.97–1.75	0.08
ACEIs/ARBs	0.73	0.54–0.97	**0.03**
Diuretics	2.20	1.62–2.98	**<0.001**
Statins	0.77	0.57–1.02	0.07
Logistic EuroSCORE (per 1 higher)	1.03	1.02–1.04	**<0.001**
Hemoglobin (per 1 g/dL higher)	0.88	0.80–0.97	**0.009**
Platelet (per 10000/μL higher)	1.00	0.989–1.002	0.20
Albumin (per 1 g/dL higher)	0.29	0.21–0.40	**<0.001**
NT-proBNP (per 100 pg/mL higher)	1.00	1.002–1.003	**<0.001**

eGFR (per 10 mL/min/1.73m^2 higher)	0.91	0.83–0.98	**0.02**
Preprocedural TTE LVEF (per 1% higher)	0.98	0.96–0.99	**<0.001**
Preprocedural TTE mean LV-Ao PG (per 1 mmHg higher)	0.99	0.980–0.995	**0.002**
Preprocedural TTE MR ≥moderate	1.81	0.92–3.56	0.08
Preprocedural TTE TR ≥moderate	4.41	2.79–6.99	**<0.001**
Preprocedural TTE TRPG (per 1 mmHg higher)	1.02	1.01–1.04	**0.001**
Valve size (per 1 mm larger)	1.08	1.01–1.15	**0.03**
LG AS (mean PG <40 mmHg)	1.92	1.42–2.58	**<0.001**
LF-LG AS (mean PG <40 mmHg, EF <50%)	1.91	1.42–2.58	**<0.001**

BMI: body mass index, COPD: chronic obstructive pulmonary disease, PAD: peripheral artery disease, CAD: coronary artery disease, ACEis: angiotensin converting enzyme inhibitors, ARBs: angiotensin Ⅱ receptor blockers, eGFR: estimated glomerular filtration rate, TTE: transthoracic echocardiography, LVEF: left ventricular ejection fraction, LV-Ao PG: left ventricular-aortic mean pressure gradient, MR: mitral regurgitation, TR: tricuspid regurgitation, TRPG: transtricuspid pressure gradient, LG AS: low gradient aortic stenosis, LF-LG AS: low-flow, low-gradient aortic stenosis. Bold font indicates statistical significance at the $p < 0.05$ level.

Supplementary Table 3. Multivariate Cox proportional hazard analyses for predictors of all-cause mortality following TAVI.

	HR	95%CI	*p*-value
Age (per 1 year older)	1.02	0.98–1.1	0.26
TAVI later than 2017	0.69	0.44–1.10	0.12
**BMI (per 1 kg/m² higher)**	**0.92**	**0.87–0.98**	**0.01**
NYHA class (per 1 higher)	1.29	0.92–1.79	0.14
Diabetes mellitus	1.51	0.90–2.55	0.12
COPD	1.26	0.81–1.98	0.31
PAD	1.02	1.00–1.04	0.06
Logistic EURO score (per 1 higher)	1.28	0.83–1.96	0.26
Hemoglobin (per 1 g/dL higher)	0.88	0.83–1.96	0.08
eGFR (per 10 mL/min/1.73m² higher)	1.00	0.99–1.01	0.74
Preprocedural TTE MR ≥moderate	0.68	0.28–1.65	0.39
Preprocedural TTE TR ≥moderate	1.67	078–3.59	0.19
Valve size (per 1 mm larger)	1.06	0.97–1.17	0.22
LF–LG AS (mean PG <40 mmHg, EF <50%)	1.14	0.75–1.74	0.53
Female AF(-)		1.0 (reference)	
**Male AF(-)**	**2.74**	**1.63–4.60**	**<0.001**
**Female AF(+)**	**3.37**	**2.01–5.66**	**<0.001**
**Male AF(+)**	**3.79**	**1.88–7.64**	**<0.001**

BMI: body mass index, COPD: chronic obstructive pulmonary disease, PAD: peripheral artery disease, eGFR: estimated glomerular filtration rate, TTE: transthoracic echocardiography, MR: mitral regurgitation, TR: tricuspid regurgitation, LF-LG AS: low-flow, low-gradient aortic stenosis. Bold font indicates statistical significance at the $p < 0.05$ level.

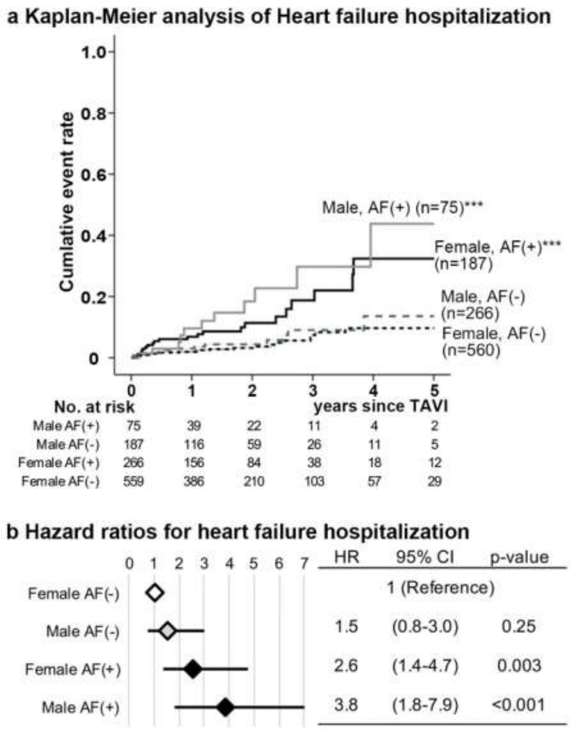

Supplementary Figure 1. Cumulative incidence (a) and hazard ratios (b) of heart failure hospitalization following TAVI in the 4 study groups according to gender and AF. (a) cumulative incidence of heart failure hospitalization in the Female AF(-), Male AF(-), Female AF(+) and Male AF(+) groups. *** indicates p<0.0001 in log-rank test of Kaplan-Meier curves compared to that of the Female AF(-) group. (b) Hazard ratios (HR), 95% confidence intervals (CI) and p-values for heart failure hospitalization following TAVI in the Male AF(-), Female AF(+) and Male AF(+) groups, when the Female AF(-) group was set as reference by Cox proportional hazard analysis using Model 2. Open, filled by gray and black rhombus indicate reference, non-significant and significantly increased risk for heart failure hospitalization, respectively.
